# Improving eHealth intervention development and quality of evaluations

**DOI:** 10.1016/S2589-7500(19)30106-2

**Published:** 2019-08-19

**Authors:** Marion Henderson, Craig Donnachie

**Affiliations:** MRC/CSO Social and Public Health Sciences Unit, University of Glasgow, Glasgow G2 3AX, UK

The systematic review and meta-analysis by Katrina Champion and colleagues^1^ in *The Lancet Digital Health* included randomised controlled trials to assess the effectiveness of school-based eHealth interventions to prevent multiple lifestyle risk behaviours among adolescents. The results showed some small but significant short-term benefits on physical activity, screen time, and fruit and vegetable intake. No improvements were found for smoking or alcohol use, or consumption of fat or sugar-sweetened beverages and snacks.

The authors were constrained by the nature of the evidence available, with the quality of the randomised controlled trials identified rated as low to very low. Below we highlight four key factors that hinder the collection and reporting of good quality evidence, and suggest how digital health intervention development and evaluation could be improved.

First, the authors could not explore the size of the effect of different intervention components because most interventions and studies did not adequately express their behavioural change techniques, thus precluding meta-regression of distinct intervention components. Process evaluations are important for examining content and context (ie, the intervention components, their mapping on the behaviour change techniques, and what works best, for whom, and why) and could have partly mitigated these limitations but were lacking in the studies included in Champion and colleagues’ review. Beyond including thorough process evaluations, we suggest future research adequately expresses behaviour change techniques and their associated components, for instance, by using Michie and colleague’s taxonomy.^2^ We also recommend that future development of interventions and evaluations should produce theories of change, to show potential pathways for change, and logic models, laying out the pathways used by a particular intervention and contextual dependencies and following the UK Medical Research Council guidance for developing and evaluating complex interventions (currently being updated).^3^

Second, of the 16 included interventions, a quarter of the randomised controlled trials expressed their comparison groups as receiving education as usual, with no information on what that comprised. Education as usual might have involved an evidence-based, face-to-face intervention that could have been more effective than an eHealth intervention,^4^ or might not have involved any intervention; therefore, interpretation of these results is not possible. Three of the interventions compared an eHealth intervention with an evidence-based, face-to-face intervention that might be more influential. So, fewer than half of interventions (44%) compared an eHealth intervention with assessment only, rather than another form of intervention. Hence, uncertainty exists about the effect size of eHealth interventions, with it possibly being underestimated. To avoid such uncertainty, we recommend following the extended CONSORT guidelines for reporting social and psychological interventions,^5^ which should lead to improved transparency of descriptions of comparison groups, although even greater emphasis on this transparency in future guidelines would be helpful.

Third, the authors could only say that any effects they found were short-lived, because to date the studies have only included short-term follow-up. Funders and evaluators should consider the benefit of funding calls that enable longer-term follow-up of eHealth interventions, and possible prospective cohort studies. These issues are probably compounded by the rapid pace of technological change and resonate with another key challenge identified within the field—ie, developing an accumulating knowledge base to guide digital health intervention development.^6^ To counter these shortcomings, we highlight the need for more substantive theoretical development to understand potentially generalisable mechanisms, including the interplay between individual factors, social norms, social networks, wider communities, and system context.^7^

Finally, many of the studies used measurement tools developed for surveillance only and might not be appropriate for measuring behavioural change. This factor was particularly notable for the use of self-reported physical activity measures, which are likely to be prone to measurement error and problems with recall, particularly when assessing physical activity for children and adolescents. Specifically, these instruments could insufficiently capture incidental or sporadic bouts of activity characteristic of younger people and children.^8^ These choices of measure cast additional doubt on the results and effect sizes of the studies. Future studies should be more explicit on the measures used in intervention settings. Only three interventions included some form of device-based physical activity assessment (ie, accelerometers). Device-based measures of physical activity might overcome some of the challenges associated with relying only on self-report, particularly when examining responsiveness to change in children and adolescents. Additionally, poor quality measures of screen time have been identified as a major methodological issue in research examining the influence of use of digital technology on adolescent health and wellbeing.^9^ We suggest a need exists to develop measurement tools that are valid, reliable, and sensitive to behavioural change for different age groups.

Beyond these issues, Champion and colleagues did not take the opportunity to suggest future research should include economic evaluation of eHealth interventions, which is increasingly important for policy makers and commissioners.^10^ A need also exists to assess health inequalities, possibly amplified by digital technology. Champion and colleagues’ Article helped highlight the lack of interventions including mobile devices and applications, given the widespread use of smartphones among adolescents, research is needed in this area.

## References

[1] Champion KE, Parmenter B, McGowan C (2019). Effectiveness of school-based eHealth interventions to prevent multiple lifestyle risk behaviours among adolescents: a systematic review and meta-analysis. Lancet Digital Health.

[2] Michie S, Richardson M, Johnston M (2013). The behavior change technique taxonomy (v1) of 93 hierarchically clustered techniques: building an international consensus for the reporting of behavior change interventions. Ann Behav Med.

[3] Craig P, Dieppe P, Macintyre S, Michie S, Nazareth I, Petticrew M (2008). Developing and evaluating complex interventions: the new Medical Research Council guidance. BMJ.

[4] Rose T, Barker M, Maria Jacob C (2017). A systematic review of digital interventions for improving the diet and physical activity behaviors of adolescents. J Adolesc Health.

[5] Grant S, Mayo-Wilson E, Montgomery P (2018). CONSORT-SPI 2018 explanation and elaboration: guidance for reporting social and psychological intervention trials. Trials.

[6] Murray E, Hekler EB, Andersson G (2016). Evaluating digital health interventions: key questions and approaches. Am J Prev Med.

[7] Greenhalgh T, Wherton J, Papoutsi C (2017). Beyond adoption: a new framework for theorizing and evaluating nonadoption, abandonment, and challenges to the scale-up, spread, and sustainability of health and care technologies. J Med Internet Res.

[8] Biddle SJH, Gorely T, Pearson N, Bull FC (2011). An assessment of self-reported physical activity instruments in young people for population surveillance: project ALPHA. Int J Behav Nutr Phys Act.

[9] Orben A, Przybylski AK (2019). The association between adolescent well-being and digital technology use. Nat Hum Behav.

[10] Petrou S, Gray A (2011). Economic evaluation alongside randomised controlled trials: design, conduct, analysis, and reporting. BMJ.

